# New Players in Metabolic Syndrome

**DOI:** 10.3390/metabo15060380

**Published:** 2025-06-09

**Authors:** Iveta Nedeva, Yavor Assyov, Vera Karamfilova, Zdravko Kamenov, Pavel Dobrev, Tsvetelina Velikova, Vlayko Vodenicharov

**Affiliations:** 1 Department of Epidemiology and Hygiene, Medical University-Sofia, Zdrave Str., 1431 Sofia, Bulgaria; v.vodenicharov@medfac.mu-sofia.bg; 2 Department of Internal Medicine, Medical University-Sofia, 1431 Sofia, Bulgaria; yavovian@abv.bg (Y.A.); verakaramfilova@abv.bg (V.K.); zkamenov@medfac.mu-sofia.bg (Z.K.); 3 Clinic of Endocrinology, University Hospital “Alexandrovska”, 1431 Sofia, Bulgaria; 4 Medical Faculty, Burgas State University “Prof. Dr. Assen Zlatarov”, 8010 Burgas, Bulgaria; dr_dobrev@abv.bg; 5 Medical Faculty, Sofia University “St. Kliment Ohridski”, 1504 Sofia, Bulgaria; tsvelikova@medfac.mu-sofia.bg

**Keywords:** metabolic syndrome, insulin resistance, carbohydrate disturbances

## Abstract

**Background/Objectives**: Metabolic syndrome (MetS) is a complex, multifaceted disorder with significant socioeconomic and public health consequences, increasingly acknowledged as a global epidemic. Fibroblast growth factor 21 (FGF-21) is known to play a vital role in metabolic regulation; however, the precise roles and interactions of free fatty acids (FFAs) and insulin in influencing FGF-21 activity under both normal and pathological conditions are not yet fully understood. Meteorin-like protein (Metrnl) is a newly identified adipokine that appears to have the potential to regulate metabolic inflammation, which is a critical pathological factor in obesity and insulin resistance. Additionally, nesfatin-1, which is widely expressed in both central and peripheral tissues, is thought to be involved in various physiological functions beyond appetite control, such as glucose homeostasis, stress response, and cardiovascular health. Recent studies have indicated that sortilin may play a role in the pathophysiology of several metabolic disorders, including type 2 diabetes mellitus. **Methods**: This investigation was a cross-sectional study involving 200 individuals with obesity, which included both metabolically healthy obese participants and those experiencing obesity along with glycemic disorders. Serum levels of FGF-21, sortilin, Metrnl, and nesfatin-1 were measured using standardized enzyme-linked immunosorbent assay (ELISA) techniques. **Results**: The results indicated that FGF-21 levels were significantly elevated in patients with metabolic syndrome (*p* < 0.001), as well as those with insulin resistance (*p* = 0.009) and dyslipidemia (*p* = 0.03). Serum Metrnl levels were notably elevated in individuals meeting the criteria for insulin resistance, with a statistical significance of *p* < 0.001. Additionally, patients experiencing carbohydrate metabolism disorders exhibited significantly higher serum sortilin levels compared to those with normal blood glucose levels, with a *p*-value of 0.003. **Conclusions**: This research highlights FGF-21, Metrnl, nesfatin-1, and sortilin as potential biomarkers involved in the development of critical aspects of metabolic syndrome.

## 1. Introduction

As a multifaceted disorder with considerable socioeconomic implications, metabolic syndrome (MetS) is recognized as a global health crisis. MetS is defined by a collection of interrelated factors that significantly increase the likelihood of developing type 2 diabetes mellitus (DMT2), coronary heart disease (CHD), and other cardiovascular atherosclerotic conditions (CVD). The main components include dyslipidemia, high arterial blood pressure (BP), and impaired glucose regulation. Nevertheless, abdominal obesity and/or insulin resistance (IR) have gained more focus as the primary indicators of the syndrome. Recent discussions have introduced additional complications to the definition of the syndrome, such as sleep apnea, non-alcoholic fatty liver disease, and chronic pro-inflammatory and pro-thrombotic states, among other anomalies.

At present, the most widely accepted definitions are those set forth by the NCEP, ATP III, and the IDF, which particularly highlight waist circumference as a key indicator of central adiposity [1,2]. In contrast, the definitions put forth by the AACE, WHO, and EGIR mainly focus on the notion of insulin resistance. The prevalence of metabolic syndrome varies significantly, ranging from 10% to 84%, influenced by various factors such as diagnostic criteria, age, gender, ethnicity, and geographic location. This condition is notably more common among individuals over the age of 60, with marked ethnic variations in its occurrence.

Consequently, research aimed at identifying biomarkers associated with the onset of metabolic syndrome could prove valuable in pinpointing risk factors or therapeutic targets for obesity, cardiovascular disease, and metabolic syndrome.

Fibroblast growth factor-21 (FGF-21) is a polypeptide consisting of 210 amino acids, predominantly produced in the liver [3]. It is also expressed to a lesser extent in skeletal muscle [4], adipose tissue, and pancreatic beta cells [4]. This protein is recognized as a novel endocrine biomarker and is part of the fibroblast growth factor family. FGF-21 plays a beneficial role as a metabolic hormone, contributing positively to the regulation of energy, lipid levels, and glucose balance in both humans and animals. Previous studies have identified significant correlations between FGF-21 levels and metabolic syndrome (MetS), as well as related disorders such as diabetes mellitus, the progression of kidney disease in diabetes [5], and coronary artery disease. However, the precise role and molecular mechanisms through which FGF-21 influences metabolic regulation remain to be fully elucidated.

Sortilin, a transmembrane glycoprotein that is part of the mammalian vacuolar protein sorting 10p domain (VPS10) family, has been identified as a novel facilitator of lipid, cytokine, and enzyme interactions that play a role in the regulation of intracellular protein transport [6]. Furthermore, it is functionally connected to various biological processes, including gene expression regulation, ossification, apoptosis, neuronal survival, tumor cell viability, and the metabolism of glucose and lipoproteins. While sortilin is predominantly a membrane-bound protein, it has been detected in minimal quantities in the bloodstream [7]. Recent studies have associated sortilin with disturbances in lipoprotein metabolism and have suggested its involvement in several disorders, including neurological, cardiovascular, and metabolic conditions such as type 2 diabetes mellitus [8]. Nonetheless, there remains a lack of research addressing sortilin levels across the full range of carbohydrate disorders, which this study aims to investigate.

Meteorin-like protein (Metrnl) is a recently identified immunoregulatory adipokine that is predominantly produced by white adipocytes, as well as activated monocytes and macrophages [9,10]. It plays a crucial role in promoting the differentiation of functional adipocytes and facilitating the browning of white adipocytes in response to thermogenic stimuli. Additionally, Metrnl helps to counteract insulin resistance and mitigate inflammatory immune responses [11]. However, there has been limited research on Metrnl in clinical contexts [12,13,14], with most data stemming from animal studies, none of which have explored the potential relationship between Metrnl and metabolic syndrome (MetS).

The neuropeptide nesfatin-1, comprising 82 amino acids, was identified as having anorexigenic properties in 2006. It is synthesized via post-translational modifications from the hypothalamic precursor protein nucleobindin-2 (NUCB2). This neuropeptide is found in various tissues, including the stomach, the pituitary gland, adipose tissue, pancreatic islet cells, and the central nervous system [15]. An increasing amount of research indicates that nesfatin-1 is involved in the regulation of metabolism due to its anti-hyperglycemic and insulinotropic effects [16]. However, the relationship between metabolic syndrome (MetS) and nesfatin-1 remains under investigation.

The primary aim of this investigation is to assess the concentrations of fibroblast growth factor 21 (FGF-21), sortilin, meteorin-like protein (Metrnl), and nesfatin-1 in individuals with obesity, regardless of the presence of carbohydrate metabolism abnormalities, within the context of metabolic syndrome. These biomarkers may serve as critical indicators of clinically relevant disruptions in metabolic processes. By delving into the roles of these factors, this study aims to uncover novel biomarkers linked to the fundamental elements of metabolic syndrome, offering the potential for a more profound understanding of the disease’s pathophysiology and its associated risk factors. Such insights could pave the way for the development of innovative diagnostic strategies and therapeutic interventions, ultimately enhancing early detection and treatment for individuals at heightened risk of metabolic disorders.

## 2. Materials and Methods

### 2.1. Study Design

This research was designed as a cross-sectional study involving 200 individuals diagnosed with obesity. The participants included both those who are obese but metabolically healthy (80 individuals—Group 1), those who exhibit obesity along with glycemic issues, specifically prediabetes (60 individuals—Group 2), and those recently diagnosed with type 2 diabetes mellitus (60 individuals—Group 3). The aim was to assess the levels of FGF-21, sortilin, Metrnl, and nesfatin-1 across these groups and to analyze their correlations with biochemical and instrumental indicators. Data collection occurred over the span of one year. This study adhered to the principles outlined in the Helsinki Declaration and received approval from the Ethics Committee of the Medical University of Sofia. Each participant provided written informed consent.

### 2.2. Inclusion and Exclusion Criteria

#### 2.2.1. Inclusion Criteria

• Age (35–74);
• BMI ≥ 30 kg/m^2^;
• Prediabetes (fasting glucose between 6.1–6.9 mmol/L, values after a 120 min oral glucose tolerance test (OGTT) between 7.8–11.0 mmol/L, and/or an HbA1c between 5.7 and 6.4%);
• Newly diagnosed diabetes (fasting glucose ≥7.0 mmol/L, values after a 120 min OGTT ≥11.1 mmol/L, and/or an HbA1c ≥6.5%), without therapy.

#### 2.2.2. Exclusion Criteria

• Liver dysfunction (liver enzymes ≥3 times above the reference range);
• Chronic kidney disease (CKD) stages III–IV;
• Heart failure (HF), classes III–IV NYHA;
• Neoplastic disease.

### 2.3. Anthropometric Parameters

• Weight, height, and waist and hip circumference;
• Weight (kg) divided by height squared (m^2^) was used to compute BMI;
• VAI = [WC/(36.68 + (1.88 × BMI)] × (TG/1.03) × (1.31/HDL) in males; VAI = [WC/(36.58 + (1.89 × BMI)] × (TG/0.81) × (1.52/HDL) for females.

### 2.4. Investigation of Glycemic Homeostasis

• Using 75 g of glucose, the oral glucose tolerance test (OGTT) was conducted by measuring the glucose and immunoreactive insulin (IRI) at 0, 60, and 120 min.
• If a patient’s HOMA index was greater than 2.5, they were deemed insulin resistant.

### 2.5. Metabolic Syndrome Was Defined by the Presence of Central Obesity (Defined as a Waist Circumference ≥80 cm for Women and ≥94 cm for Men) + Any Two of the Following Risk Factors (IDF Criteria)

- Fasting glucose ≥ 5.6 mmol/L;
- Blood pressure ≥ 130/≥ 85 mmHg;
- Triglycerides ≥ 1.7 mmol/L;
- HDL ≤ 1.03 mmol/L for men and ≤1.29 mmol/L for women.

### 2.6. Laboratory Investigation

• Serum levels of FGF-21, sortilin, Metrnl, and nesfatin-1 were measured using standard ELISA techniques. Prior to the assay, the blood samples were centrifuged and kept at −80 °C.

### 2.7. Instrumental Investigation

• Measurement of intima-media thickness (IMT) using a Panasonic Cardio Health station (Panasonic, Yogohoma, Japan);
• Cardio-ankle vascular index (VaSera system, FUKUDA DENSHI CO., Tokyo, Japan);
• Ankle-brachial index (ABI) (Elite Natus, Natus Medical Incorporated, Middleton, WI, USA);
• Assessment of the autonomic nervous system through an evaluation of sudomotor function with FDA-approved Sudoscan (Itamar Medical Ltd., Caesarea, Israel);
• Assessment of the peripheral nervous system through the neuropathy disability score.

### 2.8. Statistical Analysis

SPSS 25.0 (IBM) was the statistical software used for the analyses. The following statistical techniques were used: the Kolmogorov–Smirnov test, student’s *t*-test, descriptive analysis, and variation analysis. One-way analysis of variance between groups (ANOVA) or ++Kruskal–Wallis tests were used to analyze differences between more than two groups. With the use of multivariate logistic regression analysis, the odds ratios and 95 percent confidence intervals were calculated. *p* < 0.05 was the level of significance for rejecting the null hypothesis.

## 3. Results

This research cohort comprised 200 individuals, with an average age of 55.47 ± 9.98 years. Regarding gender, this study included 110 women and 90 men, or 55% and 45%, respectively. The participants were categorized into three distinct groups: Group 1 included 80 obese individuals without glycemic issues, Group 2 comprised 60 prediabetic participants, and Group 3 consisted of 60 patients recently diagnosed with type 2 diabetes. The initial characteristics of these four groups are detailed in Table 1. Notably, individuals with prediabetes were younger than those newly diagnosed with diabetes, while the obese participants exhibited a significantly lower waist-to-hip ratio compared to those experiencing carbohydrate disturbances.

In relation to cardiovascular risk factors, individuals with obesity and normal glucose levels exhibited reduced serum triglyceride levels and elevated HDL levels compared to those with prediabetes and diabetes (Table 2).

**Figure 1:**
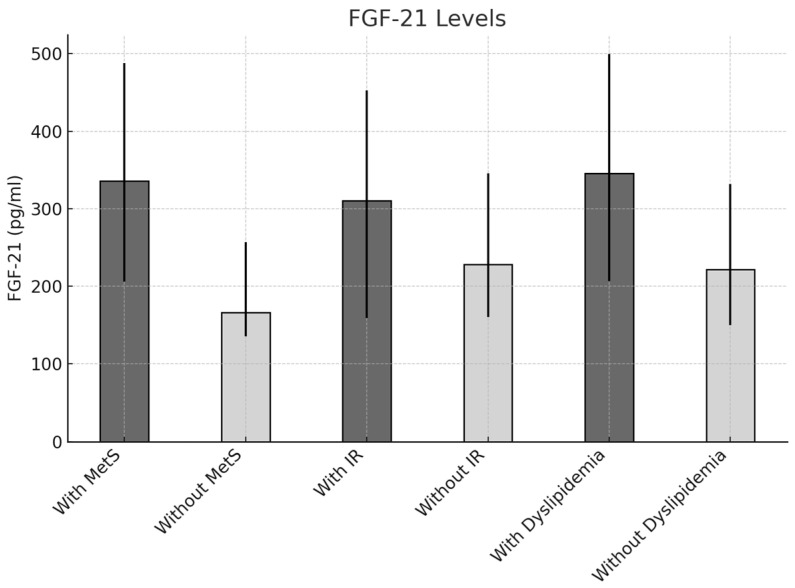
Comparison of serum FGF-21 levels based on the presence of insulin resistance, metabolic syndrome, and dyslipidemia.

**Figure 2:**
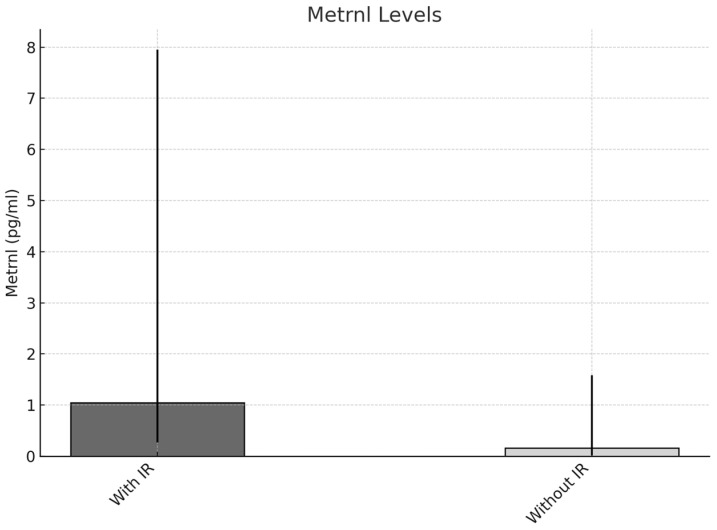
Differential serum Metrnl levels in subjects with and without insulin resistance.

The concentrations of FGF-21 were elevated in individuals diagnosed with metabolic syndrome (335.80 pg/mL [IQR = 206.22–488] compared to 166.32 pg/mL [IQR = 135.70–256.61], *p* < 0.001). Similarly, those exhibiting insulin resistance had higher levels of FGF-21 (310.73 pg/mL [IQR = 159.32–452.69] versus 228.48 pg/mL [IQR = 160.44–345.88], *p* = 0.009) and dyslipidemia (345.88 pg/mL [IQR = 206.84–499.68] compared to 221.57 pg/mL [IQR = 149.89–332.25], *p* = 0.03) (refer to Figure 1). Additionally, serum Metrnl levels were significantly higher in individuals meeting the criteria for insulin resistance (1.05 pg/mL [IQR = 0.27–7.95] versus 0.16 pg/mL [IQR = 0.02–1.58], *p* < 0.001) (refer to Figure 2).

**Figure 3:**
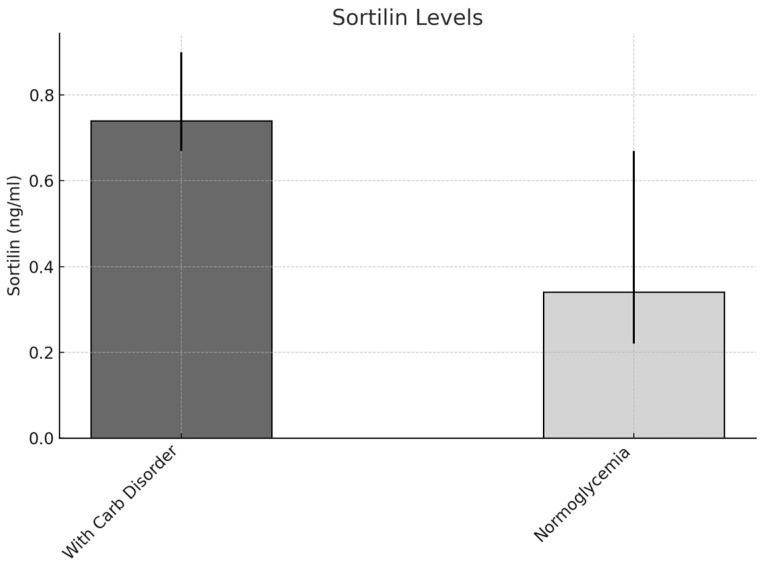
Comparison of serum sortilin levels in individuals, stratified by the presence of carbohydrate disorders.

Patients experiencing carbohydrate disorders, such as prediabetes and recently diagnosed diabetes, exhibited notably elevated serum sortilin levels when compared to individuals with normal blood sugar levels (0.74 ng/mL [IQR = 0.67–0.90] vs. 0.34 ng/mL [IQR = 0.22–0.67], *p* = 0.003) (Figure 3).

Spearman correlation analysis indicated a positive relationship between FGF-21 and various indicators of insulin resistance (IRI at 0, 60, and 120 min and HOMA) and liver enzymes (ALAT and GGT), along with the visceral adiposity index, triglycerides, and uric acid. Furthermore, a positive correlation was observed between serum nesfatin-1 levels and the waist-to-hip ratio (WHR), fasting glucose, fasting insulin, and the HOMA index during the second hour following the oral glucose tolerance test (OGTT). Metrnl also exhibited a positive correlation with several anthropometric measurements (waist circumference, WHR, and visceral adiposity index), as well as fasting insulin, the HOMA index, creatine phosphokinase (CPK), and the albumin creatinine ratio (ACR) during the first hour of the OGTT. A mildly positive association was identified between sortilin-1 levels, fasting glucose, and measurements taken during the second hour post-OGTT, as well as HbA1c% (Table 3).

We also examined the relationship between the biomarkers and various instrumental parameters related to both microvascular and macrovascular complications. A significant positive correlation was noted between the levels of FGF-21 and the intima-media thickness (IMT) of the common carotid artery [r = 0.312, *p* < 0.01], as well as between Metrnl and the cardio-ankle vascular index (CAVI) [r = 0.236, *p* < 0.05].

To assess the efficacy of FGF-21, Metrnl, and sortilin as biomarkers for differentiating individuals with metabolic syndrome, insulin resistance, carbohydrate disorders, and dyslipidemia, receiver operating characteristic (ROC) curve analysis was performed. A serum FGF-21 level of ≥269 ng/mL indicated a sensitivity of 67% and a specificity of 80% for identifying insulin resistance. Additionally, FGF-21 levels of ≥285.6 ng/mL yielded 64% sensitivity and 84% specificity for distinguishing individuals with metabolic syndrome from those who do not meet the criteria, while levels of ≥275.7 ng/mL provided a sensitivity of 68% and specificity of 65% for identifying subjects with dyslipidemia. Furthermore, a serum Metrnl threshold of ≥0.285 pg/mL emerged as a superior predictor for insulin resistance, exhibiting sensitivity and specificity rates of 73% and 71%, respectively. Individuals with serum sortilin levels of ≥0.59 ng/mL faced nearly six times the risk of carbohydrate disorders compared to those with lower levels (Table 4).

## 4. Discussion

Metabolic syndrome (MS) and its related disorders constitute a significant socioeconomic challenge worldwide, impacting individuals regardless of their socioeconomic background or ethnicity. Although its high prevalence is widely acknowledged, variations in the definitions and diagnostic criteria suggested by entities such as the World Health Organization (WHO), the Adult Treatment Panel III (ATPIII), and the International Diabetes Federation (IDF) hinder accurate evaluations of the syndrome’s true impact. Given the considerable public healthcare costs associated with managing MS and its risk factors, including obesity, hypertension, dyslipidemia, and insulin resistance (IR), it is essential to create a standardized definition. Such standardization would improve diagnostic accuracy, thereby supporting both the prevention and effective treatment of MS and its frequently linked chronic conditions, including type 2 diabetes (T2D) and cardiovascular disease (CVD). Alongside aligning diagnostic criteria, implementing early preventive measures is vital to alleviate the substantial economic burden that arises from treating these risk factors.

The present research introduces novel insights regarding the involvement of specific biomarkers, including FGF-21, Metrnl, sortilin, and nesfatin-1, in the development of major disorders associated with metabolic syndrome (MS) and their potential utility as prognostic indicators for diagnosing these conditions.

FGF-21, a hepatokine mainly synthesized in the liver, is also produced in smaller amounts by adipose tissue, skeletal muscle, the pancreas, and the thymus. Its levels rise in response to metabolic disturbances and it has been proposed as a potential biomarker for the early detection of metabolic disorders [17,18].

Originally identified as a beneficial cytokine involved in metabolic regulation, FGF-21 promotes glucose uptake independently of insulin. Pre-clinical studies have demonstrated that FGF-21 increases the mRNA expression of glucose transporter 1 (GLUT1) in adipocyte membranes, thereby facilitating glucose absorption in insulin-resistant models, such as ob/ob mice. Furthermore, systemic administration of FGF-21 has been shown to significantly reduce both serum triglyceride and glucose levels while enhancing lipoprotein profiles in genetically diabetic primates. However, despite its favorable metabolic effects, elevated serum FGF-21 levels have been consistently observed in obese individuals with metabolic syndrome (MS) compared to healthy individuals [19,20] These levels tend to rise progressively as glycemic control worsens, transitioning from normal glucose tolerance to prediabetes and type 2 diabetes mellitus, and are notably elevated in various cardiometabolic conditions, including obesity, MetS, T2DM, coronary artery disease, and non-alcoholic fatty liver disease (NAFLD) [21]. Our findings corroborate these observations, revealing increased circulating FGF-21 levels in individuals with metabolic syndrome, insulin resistance, and dyslipidemia. Additionally, this study highlighted a correlation between FGF-21 levels and insulin resistance indicators, such as fasting insulin, measurements from the first and second hours of the oral glucose tolerance test (OGTT), and the HOMA index, as well as triglycerides, HDL, VLDL, uric acid, and GGT. A recent investigation [22] identified that supraphysiological levels of free fatty acids (FFAs) induced by lipid-heparin infusion were associated with elevated serum FGF-21 concentrations, alongside hyperinsulinemia. This finding implies that increased FFAs, which significantly contribute to the onset of insulin resistance through enhanced lipolysis, may act as a primary stimulus for the upregulation of serum FGF-21 levels.

Although the involvement of FGF21 in atherosclerosis has been established through genetic knockout mouse studies, its correlation with carotid intima-media thickness (IMT) in humans is not well defined. In our research, we have, for the first time, identified a positive association between serum FGF-21 levels and IMT in obese individuals experiencing early carbohydrate metabolism disorders, specifically prediabetes and newly diagnosed diabetes.

Our results reinforce the utility of FGF21 as a biomarker, as its serum levels were found to rise in direct relation to the severity of metabolic disorders. Notably, subjects with FGF-21 levels equal to or exceeding 285.6 ng/mL exhibited an 11.4-fold increased risk of metabolic syndrome in comparison to those with lower levels. Furthermore, FGF-21 showed predictive capabilities for insulin resistance and dyslipidemia; serum concentrations ≥269 ng/mL were linked to an 8-fold heightened risk of insulin resistance, while levels of ≥275.7 ng/mL were connected to a 4.02-fold increased risk of dyslipidemia.

Research regarding the meteorin-like protein (Metrnl), an adipomyokine involved in metabolic regulation, has grown; however, its classification as beneficial or harmful remains ambiguous. Investigations concerning patients with type 2 diabetes mellitus (T2DM) have yielded particularly inconsistent results. For instance, while studies by Lee et al. [23] and Zheng et al. [24] reported reduced serum Metrnl levels in newly diagnosed T2DM patients, other research noted increased Metrnl concentrations in those with established T2DM [25,26].

Given the limited number of studies examining the relationship between type 2 diabetes mellitus and serum Metrnl levels, resolving the conflicting evidence in the literature remains a challenge. Pre-clinical evidence in mice suggests that Metrnl expression in adipocytes may improve insulin sensitivity via the activation of the peroxisome proliferator-activated receptor gamma (PPARγ) pathway, which is crucial for managing insulin resistance and adipocyte differentiation [27]. Conversely, a study on human adipocytes indicated that Metrnl overexpression may inhibit PPARγ expression, potentially leading to hyperinsulinemia and insulin resistance [28]. These findings imply that elevated circulating Metrnl levels could, under specific conditions, exacerbate insulin resistance and heighten the risk of T2DM—an interpretation that aligns with our study’s outcomes. Supporting this theory, our results showed significantly elevated serum Metrnl levels in individuals with insulin resistance, revealing a positive correlation between serum Metrnl levels and both fasting insulin and insulin levels recorded during the initial hour of the oral glucose tolerance test (OGTT). Additionally, we assessed serum Metrnl levels for the first time in patients with early-stage carbohydrate metabolism disorders, namely prediabetes.

Considering the high expression of Metrnl in white adipose tissue, its serum levels are likely to correlate with anthropometric measurements such as body mass index (BMI) and waist circumference (WC). Our study findings indicated a significant positive correlation between serum Metrnl levels and key anthropometric indicators associated with visceral obesity, including waist circumference, waist-to-hip ratio, and the visceral adiposity index. Notably, we revealed a novel positive relationship between Metrnl, creatine phosphokinase, and the cardio-ankle vascular index. These results provide a foundation for hypothesizing that Metrnl may play a critical role in the pathogenesis of endothelial dysfunction and consequently in the progression of cardiovascular diseases.

To the best of our knowledge, this study is the first to recognize Metrnl as a potential predictor of insulin resistance. Serum levels ≥0.285 pg/mL were associated with an approximately 6.5-fold increased risk of developing this condition.

The significant role of nesfatin-1 in glucose and insulin metabolism has been emphasized in various studies; however, the results concerning its connection to diabetes and insulin resistance show considerable variability. For example, research by Su et al. [29] indicated that administering nesfatin-1 to rats led to a notable reduction in plasma glucose levels, implying that its anti-hyperglycemic effects could be linked to insulin signaling pathways, although the precise mechanisms remain unclear. Some clinical investigations have reported lower nesfatin-1 levels in individuals with type 2 diabetes mellitus (T2DM), while higher concentrations have been noted in patients with type 1 diabetes mellitus when compared to healthy subjects [30]. Conversely, other studies have found increased nesfatin-1 levels in individuals with T2DM and those with impaired glucose tolerance, revealing positive correlations with fasting blood glucose (FBG), plasma insulin, glucose levels measured two hours post-load, and HOMA-IR scores [31]. Additionally, intravenous glucose infusion has been shown to significantly increase baseline nesfatin-1 levels in healthy adults [32]. Our findings align with these results, as we also found a positive correlation between nesfatin-1 and FBG, fasting insulin, and the first hour after the oral glucose tolerance test (OGTT), along with the HOMA index and waist-to-hip ratio.

Sortilin is a versatile regulatory protein that serves as a receptor for various neurotrophic factors and neuropeptides. Beyond its primary role as a receptor, sortilin functions as a co-receptor in conjunction with multiple signaling molecules, including cytokine receptors, tyrosine kinase receptors, G-protein-coupled receptors, and ion channels. Its expression is notably abundant in metabolically active tissues such as the liver, brain, skeletal muscle, and adipose tissue, underscoring its potential involvement in diverse physiological and metabolic processes [33]. Type 2 diabetes mellitus (T2DM), frequently associated with insulin resistance, has been linked to changes in circulating sortilin levels and genetic variations in the sortilin 1-related VPS10 domain-containing receptor 1 (SORC1) gene [34,35]. For instance, Demir et al. noted reduced serum sortilin levels and a negative correlation between sortilin concentrations and insulin resistance in a group of 75 newly diagnosed T2DM patients [36]. In contrast, Oh et al. reported increased serum sortilin levels in statin-naïve T2DM patients with concurrent coronary artery disease. Their study also found that individuals with the highest circulating sortilin concentrations had elevated HbA1c and fasting glucose levels [37]. Our results are consistent with those reported by Oh et al., as we also observed significantly raised serum sortilin levels in individuals experiencing early disturbances in carbohydrate metabolism, including prediabetes and newly diagnosed type 2 diabetes. Furthermore, a positive correlation was noted between serum sortilin concentrations and both fasting glucose levels and glucose levels measured two hours following the OGTT. Importantly, to our knowledge, this is the first study to show that serum sortilin levels of ≥0.59 ng/mL are associated with approximately a sixfold increased risk of disturbances in carbohydrate metabolism. These conflicting results suggest that sortilin expression may be affected by the stage of the disease, comorbid conditions, or treatment status.

### 4.1. Clinical Significance

In the present study, threshold levels of FGF-21, Metrnl, and sortilin were identified for the first time, associated with the presence of insulin resistance and dysglycemia. Additionally, a novel and significant correlation was established between the serum levels of these markers and certain instrumental indicators related to early endothelial dysfunction.

### 4.2. Limitations of This Study

The limitations of this study include its cross-sectional design, a relatively small sample size, and the absence of mechanistic data; therefore, these results should be viewed cautiously.

## 5. Conclusions

This research identified FGF-21, Metrnl, nesfatin-1, and sortilin as potential biomarkers associated with the development of critical elements of metabolic syndrome. Beyond their role in pathogenesis, these biomarkers could act as valuable predictors for the early detection of individuals who are at a heightened risk for disorders related to carbohydrate metabolism. To confirm these relationships and evaluate the clinical utility of these biomarkers in the stratification of metabolic risk, further studies involving larger and more diverse populations, as well as employing prospective, mechanistic, and longitudinal methodologies, are essential.

## Figures and Tables

**Table 1:** Anthropological features of the research cohorts.

	Obesity Group 1	Prediabetes Group 2	Diabetes Group 3	1/2	1/3	2/3
***n***	80	60	60			
**Age years**	53.54 ± 9.09	53.74 ± 11.65	55.92 ± 11.02	ns	ns	***p* < 0.05**
**BMI (kg/m^2^)**	34.65 ± 3.10	35.85 ± 4.97	34.69 ± 5.56	ns	ns	ns
**WHR**	0.90 ± 0.09	0.91 ± 0.10	0.96 ± 0.16	***p* < 0.05**	ns	***p* < 0.05**
**WSR**	0.64 ± 0.06	0.65 ± 0.07	0.65 ± 0.07	ns	ns	ns
**Waist cm**	104.58 ± 10.38	106.17 ± 11.68	108.36 ± 13.96	ns	ns	ns

Note. N—number; BMI—body mass index; WHR—waist-to-hip ratio; WSR—waist-to-stature ratio; ns—non-significant.

**Table 2:** Heart-related risk factors among the groups.

	Obesity Group 1	Prediabetes Group 2	Diabetes Group 3	1/2	1/3	2/3
**N**	80	60	60			
**SBP (mmHg)**	130 [120–139.5]	120 [112.5–130]	130 [120–140]	ns	ns	ns
**DBP (mmHg)**	80 [70–90]	80 [72.3–80]	80 [72.3–87.5]	ns	ns	ns
**Tchol (mmol/L)**	5.33 (1.21)	5.29 (1.08)	5.55 (1.26)	ns	ns	ns
**LDL (mmol/L)**	3.33 (1.05)	3.20 (0.99)	3.23 (1.10)	ns	ns	ns
**HDL (mmol/L)**	1.35 (0.30)	1.24(0.31)	1.08 (0.30)	***p* < 0.05**	***p* < 0.05**	***p* < 0.05**
**TG (mmol/L)**	1.29 [0.87–1.67]	1.55 [1.28–2.07]	1.94 [1.38–1.94]	***p* < 0.05**	***p* < 0.001**	***p* < 0.05**
**Hypertesion**	76.3%	85.5%	84.7%	ns	ns	ns
**Smoking %**	26.6%	18.0%	33.9%	ns	ns	ns
**Dyslipidemia %**	46.3%	62.9%	79.3%	ns	***p* < 0.05**	ns

Note. Normally distributed data are presented using means (±SD). Skewed distributed data are presented as medians [IOR25–IOR75]; ns—non-significant. To the right are the *p*-values of Kruskal–Wallis tests (if the distribution was not normal) and ANOVAs (if the distribution was normal). Tchol—total cholesterol; TG—triglycerides; SBP—systolic blood pressure; DBP—diastolic blood pressure.

**Table 3:** Spearman correlations of circulating serum FGF-21, nesfatin-1, Metrnl, and sortilin with anthropometric and laboratory parameters of the research cohort.

Variable	FGF21	NEFA	METRNL	SORT
Age	0.112	−0.010	−0.014	−0.039
BMI	0.068	0.088	0.101	0.041
WC (cm)	0.176	0.175	0.288 *	0.158
HC (cm)	0.029	−0.112	0.010	0.091
WHR	0.093	0.297 *	0.353 **	0.189
WSR	0.099	0.208	0.176	0.078
VAI	0.330 **	0.022	0.355 **	0.117
FAT (%)	0.064	0.048	0.145	0.031
SBP (mmHg)	0.003	0.011	0.068	0.047
DBP (mmHg)	−0.007	0.075	0.125	0.013
Tchol (mmol/L)	0.042	0.048	0.021	0.063
VLDL(mmol/L)	0.296 **	0.021	0.127	0.189
HDL (mmol/L)	−0.289 *	−0.002	−0.032	−0.157
LDL (mmol/L)	0.044	0.060	0.014	0.071
TG (mmol/L)	0.296 **	0.015	0.128	0.187
Gluc 0 min (mmol/L)	0.119	0.282 *	0.190	0.393 **
Gluc 60 min (mmol/L)	0.047	0.227	0.081	0.126
Gluc 120 min (mmol/L)	0.072	0.391 **	0.082	0.351 **
IRI 0 min (mU/L)	0.295 *	0.238 *	0.272 *	0.142
IRI 60 min (mU/L)	0.318 **	0.138	0.285 *	0.134
IRI 120 min (mU/L)	0.276 *	0.242	0.122	0.186
ASAT (U/L)	0.098	−0.113	0.099	0.174
ALAT (U/L)	0.235 *	−0.110	0.119	0.189
GGT (U/L)	0.418 ***	−0.009	0.074	0.239 *
Creat (mkmol/L)	0.124	−0.142	−0.156	0.048
Uric acid (mkmol/L)	0.411 ***	−0.004	0.088	0.028
HOMA-IR	0.270 *	0.256 *	0.268 *	0.100
CPK (U/L)	0.049	−0.110	0.272 *	−0.030
HbA1c%	0.166	0.136	0.181	0.497 **
ACR (mg/mmol)	0.155	−0.024	0.389 *	0.082
eGFR	−0.098	0.175	0.024	0.018

Note. NEFA—nesfatin-1; Metrnl—meteorin-like protein; SORT—sortilin; BMI—body mass index; WC—waist circumference; HC—hip circumference; WHR—waist-to-hip ratio; WST—waist-to-stature ratio; VAI—visceral adiposity index; Gluc 0, 60, 120 min—glucose 0, 60, 120 min within the oral glucose tolerance test; IRI 0, 60, 120 min—immunoreactive insulin within the oral glucose tolerance test; HOMA-IR—homeostatic model assessment of insulin resistance; ASAT—aspartat amino transferase; ALAT—alanine amino transferase; GGT—gamma glutamyl transferase; SP—systolic pressure; DP—diastolic pressure; CPK—creatinine phosphokinase; ACR—albumin/creatinine ratio. * *p* < 0.05; ** *p* < 0.01; *** *p* < 0.001.

**Table 4:** Odds ratios (ORs) and 95% confidence intervals (Cls) for distinguishing pathology state (metabolic syndrome, insulin resistance, carbohydrate disorders, and dyslipidemia).

				95% CI		
Pathology State	Biomarker	Value	ORs	Upper limit	Lower limit	*p*
Metabolic syndrome	FGF-21	≥285.6/<285.6	11.400	3.001	43.307	<0.001
Insulin resistance	FGF-21	≥269/<269	8.000	2.272	28.170	0.001
Insulin resistance	METRNL	≥0.285/<0.285	6.400	1.859	22.036	0.003
Dyslipidemia	FGF-21	≥275.7/<275.7	4.024	1.566	10.336	0.004
Carbohydrate disorders	Sortilin	≥0.59/<0.59	5.784	1.515	10.182	0.028

## Data Availability

Data unavaible due to privacy or ethical restrictions.
